# Cannabis treatment outcomes among legally coerced and non-coerced adults

**DOI:** 10.1186/1471-2458-7-111

**Published:** 2007-06-14

**Authors:** Jan Copeland, Jane C Maxwell

**Affiliations:** 1 National Drug and Alcohol Research Centre, University of New South Wales, Sydney, 2052, Australia; 2 Addiction Research Institute, Center for Social Work Research, University of Texas at Austin, USA

## Abstract

**Background:**

Treatment seeking for cannabis dependence in general, and particularly the number of criminal justice referrals to cannabis treatment, has increased over the past decade. This study aims to compare the characteristics, psychosocial functioning and treatment outcome of those legally coerced into cannabis treatment compared to those entering treatment without legal coercion.

**Methods:**

This study is a retrospective audit of the administrative clinical records of 27,198 adults presenting to public Texas treatment programs with cannabis as their primary drug problem between 2000 and 2005.

**Results:**

Of the 69% legally coerced into treatment, there was less psychological distress and greater likelihood of having completed treatment compared with non-coerced clients. Participants who were legally coerced into treatment were also more likely to have received less intensive forms of treatment and to have not used cannabis in the month prior to 90-day post-treatment follow-up.

**Conclusion:**

More public health information is needed on cannabis dependence and increased availability of subsidised early and brief interventions in a variety of primary health care settings would reduce the late presentations of the more severely impaired voluntary clients. The limitations of this dataset are discussed.

## Background

Substance use and related problems have presented an increasing challenge for most countries over the past few decades [1]. Cannabis is the most commonly used of the illicit drugs internationally [2]. A substantial proportion of cannabis users develop cannabis-related problems, including abuse and dependence [3,4]. Recent analyses comparing the past year prevalence of cannabis use disorder in large US epidemiological studies between 1991 and 2002 found significant increases from 1.2% to 1.5% with disproportionate increases in African-American and Hispanic men and in African-American females [5]. There are estimated to be 4.1 million Americans aged 12 years or over who abused or were dependent on cannabis, accounting for around 60% of the illicit drug use disorders overall [6].

The scientific evidence on the nature and treatment of cannabis use disorder, however, has only developed over the past decade. While there is a small but growing literature of the efficacy of interventions for cannabis use disorder, there are few reports on the effectiveness of cannabis treatment as it is provided within publicly-funded treatment agencies. While dependence is the most common harm associated with cannabis use, there is also a clear relationship between substance use and crime [7]. As a result there is a growing concern to have a more effective response to drug-related crime than imprisonment and other solely legal justice responses [8]. In the US and Australia, and increasingly across the developing and developed world, drug courts have been introduced to divert drug-using offenders away from prison into programs involving drug-testing, treatment, supervision and court-mandated sanctions for non-compliance [9,10]. The ethical issues associated with legally coerced treatment are an important factor in the formation of public policy [11].

In general, coercion has been shown to be a predictor of successful treatment outcome [12,13]. Research into the effectiveness of coerced substance use treatment has been conducted over the past three decades and has yielded an inconclusive pattern of results [7]. Some studies have suggested that legally coerced and non-coerced treatment clients do not differ in treatment outcome [14] while other studies have reported that non-coerced clients have superior outcomes [15].

A recent study on aspects of legal coercion and motivation found that legal coercion was positively associated with readiness to change [16]. A UK study using routine treatment monitoring data, however, has reported that legal coercion was associated with higher levels of treatment drop-out [17]. The contrariety of findings on this important social and legal question highlights the need for further examination of the impact of legal coercion on illicit drug treatment outcome. There have been no studies on correlates and outcomes of legal coercion that have focused on the treatment of the most common illicit drug, cannabis.

This study aims to examine the characteristics, treatment completion and 90-day treatment follow-up outcomes of legally coerced versus non-coerced clients presenting to a large, publicly-funded treatment system in Texas, USA from 2000–2005 with cannabis as their principal drug of concern.

## Methods

This study examined a total of 27,198 unduplicated administrative records on adult Texans with cannabis as the primary problem who were treated in federal and state-funded programs between January 1, 2000 and December 31, 2005. Another 217 records of clients who were readmitted during this time period were deleted from the dataset, with only their first admission included in the analyses. These clients represented 11% of all alcohol and drug clients who entered treatment during this time period (32% of all clients had a primary problem with alcohol, 21% with crack cocaine; and 11% with heroin).

The dataset was extracted from the Behavioral Health Integrated Provider System (BHIPS), which is an Internet-based system of administrative records developed by the Texas Department of State Health Services (DSHS), formerly the Texas Commission on Alcohol and Drug Abuse. BHIPS provides record keeping, data sharing within a service network, and supports state and federal administrative data reporting requirements, including the federal Treatment Episode Data System (TEDS). Reimbursement for services is tied to submission of the required client data forms.

Data collected at admission by the treatment programs primarily reflects the living and economic status of the client at that time, as well as substance use of the client before admission, and the number of days in the last month that the client experienced any of the six domains of the Addiction Severity Index (ASI) [18]. Reports are also collected on the client's condition at discharge and at follow-up 90 days after the last service. DSHS requires that follow-up contact be made in person or by telephone. If the client cannot be located, information about the client may be obtained from family members, case worker, parole or probation officers, or other persons (provided the client had given written permission to make such contacts).

BHIPS also collects information from the treatment programs on Diagnostic and Statistical Manual of Mental Disorders IV (DSM-IV), although there are inconsistencies in the extent of reporting the diagnoses since programs without staff trained to do the DSM diagnosis do not report those data.

### Analyses

Means are reported for continuous data and categorical variables are described in percentages. When comparisons between non-coerced and coerced clients are made, t-tests are used for comparisons between normally distributed continuous data and χ^2 ^for categorical data. Binominal odds ratios were calculated using SAS v9 PROC GENMOD, which can model categorical, ordinal, and continuous responses. Variables that approached a significance of *p *< .10 were included in logistic regression analyses to identify independent predictors of being coerced to treatment, completing treatment, and being abstinent at 90-day follow-up. Because clients within a local program might have characteristics more similar to each other than those randomly selected from other programs, the Generalized Estimating Equation (GEE) model was used to account for the variation in user characteristics due to treatment programs. Significance was set at *p *< 0.05 using the GEE parameter estimates. No research on humans was carried out by the authors. The secondary analysis of this administrative dataset was approved by the Institutional Review Board of the University of Texas at Austin.

## Results

### Characteristics of Non-coerced and Coerced Clients at Admission

Texas treatment programs which are funded by DSHS are public community mental health centers or non-profit private entities and represent approximately 72% of treatment services in Texas. They are licensed to meet health and safety standards and are contracted to provide specific treatments which are reimbursed based on units of service. Eligibility is based on clinical and financial need. Thus, individuals with the means to enter private treatment are not included in this dataset.

Some 69% of cannabis admissions were involved with the criminal justice system, including those who had a legal status (awaiting trial, diverted to treatment, on probation, parole, or in jail) and those referred to treatment from a criminal justice source (probation, parole, police, or courts). Referral sources for non-coerced admissions included self (20%), social services or protective services (26%), community mental health centers (8%), family or friends (6%), or local councils on alcohol and drugs (6%).

Some 47% of the clients entered programs which reported DSM-IV diagnoses, and of those diagnosed, 84% of the coerced and 69% of the voluntary clients had no condition on Axis I or Axis II. However, some 7% of the coerced and 14% of the voluntary clients had a mood depressive disorder (χ^2 ^284.5, *p *< .0001) and another 4% of coerced and 9% of voluntary clients were diagnosed with bipolar disorders (χ^2 ^283.7, *p *< .0001). At admission, 6% of all-coerced and 13% of all non-coerced patients were prescribed anti-depressant or anti-anxiety medications (χ^2 ^400.70, *p *< .0001) with less than 2% of clients being prescribed any other medication at admission.

Of those patients who received a substance-related DSM-IV diagnosis, 24% were diagnosed as cannabis abusers, 55% were cannabis dependent, and 9% were polysubstance dependent. Fifty-five percent of the coerced clients and 54% of the voluntary clients met the criteria for cannabis dependence (*p *= 0.1098), 26% of the coerced and 18% of the non-coerced met the criteria for cannabis abuse (*p *< .0001), and 8% of the coerced and 12% of the non-coerced met the criteria for polysubstance dependence (*p *< .0001),

These voluntary or non-coerced referrals, as compared to coerced clients, entered treatment with more days of problems in the past month as measured on the six domains of the ASI, they were more likely to be homeless, to have sought care for themselves at hospitals or emergency rooms at least once in the past year, to have used cannabis daily in the 6 months prior to admission, and to have been placed on medication for depression or anxiety problems at admission (Table 1).

**Table 1:** Characteristics of Clients at Admission to Treatment with a Primary Problem with Cannabis. Based on Their Legal Status and Odds Ratios Predicting Legally Coerced Status at Admission: 2000–2005

	**Voluntary**	**Coerced**		**Multivariate**			
						**95% CI**	
			p value	**Odds Ratios**	Pr>Z	**Lower**	**Upper**
Risk Factor	n = 8422	n = 18776		n = 24622			
Average Age	28.4	26.4	***	0.97	***	0.96	0.98
Male	46.8%	72.2%	***	2.35	***	2.04	2.71
Black	25.9%	28.7%	***	1.10		0.71	1.71
White	48.2%	39.8%	***	1.00			
Hispanic	24.1%	29.8%	***	0.96		0.61	1.52
Employed at Admission	22.2%	45.4%	***	2.13	***	1.84	2.45
Placed on Medication at Admission	12.8%	5.7%	***	0.87		0.71	1.05
1 or More Past Year ER/Hospital Visits	39.8%	23.2%	***	0.92	***	0.88	0.95
Homeless	8.5%	1.6%	***	0.28	***	0.21	0.37
Days of Health Problems	4.3	2.6	***	1.01	***	1.00	1.01
Days of Employment Problems	11.3	7.3	***	1.00		0.99	1.01
Days of Family Problems	10.2	4.8	***	0.98	***	0.98	0.99
Days of Social Problems	6.8	3.4	***	1.00		0.99	1.01
Days of Psychological Problems	10.7	5.0	***	0.98	***	0.98	0.99
Days of Drug/Alcohol Problems	11.6	6.7	***	0.99	**	0.99	1.00
Used Daily in Last 6 Months	47.4%	33.0%	***	0.68	***	0.61	0.76

*p =< .05,

**p =< .01

***p =< .0001

To determine which demographic and psych-social functioning characteristics best predict criminal justice status, binominal and multinominal logistic regression models were constructed using referral status (1 = coerced and 0 = non-coerced admission). As shown in Table 1, being male, employed at admission, having health problems not related to substance use, and being younger predicted being coerced into treatment, while being homeless, seeking care in the emergency room or hospital at least once in the past year, using daily, and having more days of family, psychological, or alcohol or drug problems in the month prior to admission as measured on the ASI predicted being a voluntary or non-coerced client.

Some 75% of the clients who entered treatment with a primary problem with cannabis received outpatient services, with 20% receiving residential services and 3% receiving detoxification services. Voluntary clients were more likely to receive detoxification (5% versus 2%, χ^2 ^166.7, *p *< .0001) and residential services (36% versus 18%, χ^2 ^979.9, *p *< .0001), and less likely to be in outpatient care (64% versus 80%, χ^2 ^862.8, *p *< .0001).

### Characteristics of Non-coerced and Coerced Clients Who Completed Treatment

At discharge, 34% of non-coerced and 42% of coerced patients successfully completed the treatment service for which they were enrolled and they were either discharged or referred to an additional level of treatment at another location (χ^2 ^136.56, *p *< .0001). Another 17% of non-coerced and 21% of coerced were discharged because of violation of program rules (χ^2 ^48.50, *p *< .0001) and 12% of non-coerced and 6% of coerced left against program advice (χ^2 ^302.9, *p *< .0001). Seventy-five percent of the non-coerced and 71% of the coerced clients were abstinent in the last month of their treatment before discharge (χ^2 ^31.05, *p *< .0001).

The average length of stay from admission to date of the last face-to-face treatment contact was 70 days for coerced clients, as compared to 57 days for non-coerced clients (*p *< .0001). Coerced clients stayed in residential longer (31 days versus 28 days, *p *< .0001) and in outpatient longer (78 days versus 73 days, *p *< .0001).

Since coerced clients were more likely to complete treatment, data were analyzed to determine which variables predicted completing treatment. A binominal model was constructed using the six ASI variables, length of stay in treatment, number of family and friends involved in the treatment process, abstinence in the last month of treatment, being in residential treatment, and number of 12-Step meetings attended in the last month of treatment. Those variables which were significant were then included in multinominal models for coerced and non-coerced clients (1 = completed treatment and 0 = non-completer). As Table 2 shows, for both groups, being abstinent in the month prior to discharge, having more family and friends involved during treatment, attending more 12-step meetings during the last month of treatment, and a longer length of stay were significant factors in predicting treatment completion, while having more days of psychological problems in the month before admission predicted non-completion.

**Table 2:** Multivariate Prediction of Treatment Completion for Legally Coerced and Voluntary Clients with a Primary Problem with Cannabis: 2000–2005

	**Criminal Justice Admissions**				**Voluntary Admissions**			
		n = 13822				n = 5799		
			**95% CI**				**95% CI**	
**Risk Factor**	Odds Ratios	Pr>Z	**Lower**	**Upper**	Odds Ratios	Pr>Z	**Lower**	**Upper**
Days of Psychological Problems at Admission	0.99	***	0.98	0.99	0.99	***	0.98	0.99
Length of Stay	1.01	***	1.00	1.01	1.01	***	1.01	1.01
Family & Friends Involved in Treatment Process	1.18	***	1.10	1.27	1.13	**	1.06	1.20
Abstinent Last 30 Days of Treatment	7.95	***	6.58	9.61	5.57	***	4.41	6.78
12-Step Meetings Attended in Last 30 Days of Treatment	1.09	***	1.07	1.11	1.07	***	1.06	1.08

*p =< .05

**p =< .01

***p =< .0001

### Status of Clients at 90 day Follow-up

Information on status of clients 90 days after last treatment encounter was obtained on 68% of the clients. Contact was more likely to be made with coerced clients (75% versus 66%, χ^2 ^179.6, *p *< .0001). Of the clients, 84% of coerced and 77% of the non-coerced clients had not used cannabis in the month prior to follow-up (χ^2 ^77.2, *p *< .0001).

Binominal models were constructed to predict past-month abstinence at follow-up (1 = no use and 0 = use) using number of months employed since discharge, whether or not the client was living in a household where he or she was exposed to alcohol abuse or drug use, number of arrests since discharge, number of emergency room or hospital visits since discharge, all the ASI problem indices except days of alcohol or drug problems, number of 12-Step meetings attended in the month prior to follow-up, and whether or not the client had been in residential treatment. Those variables which were significant were then included in multinominal models for coerced and non-coerced clients (Table 3).

**Table 3:** Multivariate Prediction of Past Month Abstinence from Cannabis at 90 Day Follow-Up for Legally Coerced and Voluntary Clients: 2000–2005

	**Criminal Justice Admissions**				**Voluntary Admissions**			
		n = 7822				n = 2721		
			**95% CI**				**95% CI**	
**Risk Factor**	**Odds Ratios**	**Pr>Z**	**Lower**	**Upper**	**Odds Ratios**	**Pr>Z**	**Lower**	**Upper**
Living in Household Where Exposed to Alcohol Abuse or Drug Use	0.15	***	0.11	0.21	0.10	***	0.07	0.15
Arrests Since Discharge	0.93	*	0.86	1.00	0.75		0.48	1.15
Days of Employment Problems at Follow-up	0.97	***	0.96	0.98	0.97	**	0.95	0.99
Days of Family Problems at Follow-Up	0.96	***	0.95	0.98	0.97	**	0.95	0.99
Days of Social Problems at Follow-Up	0.99		0.97	1.03	0.95	***	0.94	0.97
Days of Psychological Problems at Follow-Up	0.95	***	0.94	0.96	0.97	**	0.95	0.99
12-Step Meetings Attended in Last 30 Days	1.13	***	1.09	1.17	1.08	**	1.04	1.13
Residential Treatment	0.86		0.63	1.16	0.58	**	0.44	0.78

*p =< .05

**p =< .01

***p =< .0001

For both groups, living in situations where they were exposed to alcohol abuse or drug use and having more days of employment, family, or psychological problems in the month prior to follow-up predicted not being abstinent at follow-up, while attending more 12-Step meetings in the previous month predicted past-month abstinence for both groups. For coerced clients, cannabis use at follow-up was also predicted by more arrests since discharge, while for voluntary clients, having been in residential treatment and having more days of social problems in the month before follow-up predicted cannabis use.

Overall, clients admitted to residential services were more impaired than those admitted to outpatient services, yet they were more likely to be abstinent in their last month of treatment (87% versus 67%, χ^2 ^870.2, *p *< .0001) and to complete treatment (70% versus 53%, χ^2 ^578.4, *p *< .0001). But they were less likely to be abstinent at follow-up (34% versus 44%, χ^2 ^207.8, *p *< .0001).

## Discussion

This study has found that individuals in state-funded alcohol and other drug services in Texas who were coerced into treatment (69%) were less impaired and more likely to have completed treatment (42%), and be abstinent from cannabis at follow-up (84%) than their non-coerced peers.

At admission, clients who voluntarily entered treatment were less likely to be employed, to have sought care in an emergency room or hospital in the past year, to have used cannabis daily in the six months prior to admission, and to report more days of family, psychological, and drug and alcohol problems

This study found that 55% of all the clients met the criteria for cannabis dependence, a number close to the 60% findings of an Australian study of individuals diverted by police after arrest for cannabis-related offences [19]. Legally coerced clients were less impaired at admission, were more likely to stay in treatment longer and to complete treatment, and were less likely to leave against program advice but were more likely to be asked to leave as a result of rule violations. These findings are in contrast to a recent UK study that reported coerced clients were more likely to drop out of treatment [17]. Participants in that study who were legally coerced into treatment were also more likely to receive less intensive forms of treatment (outpatient counseling), to be contacted at follow-up, and to have not used in the month prior to that contact. The authors concluded that their coerced status contributed to their longer length of stay so that they were more likely to have successfully completed outpatient treatment. The differences in the study outcomes may be accounted for by differences in illicit drug use. The UK study does not report by principal drug of concern but appears to focus on injecting drug users [17]. These findings highlight the importance of assessing treatment outcome by principal drug of concern.

Having more days of psychological problems was a predictor of voluntary status at treatment admission, a predictor of non-completion of treatment, and a predictor of cannabis use at follow-up, regardless of legal status. Although the level of co-occurring disorders may be underreported in this dataset, the diagnoses of depression and anxiety, particularly among the voluntary clients, and the prescribing of medications for these conditions provides evidence that treatment of co-occurring conditions would be a factor in increasing positive treatment outcomes.

Family and friends affected treatment outcomes both positively and negatively. Having more days of family problems at admission was a predictor of voluntary status and it was a predictor of cannabis use at follow-up for both non-coerced and coerced clients. Individuals who had more family and friends involved in their treatment process were more likely to complete treatment, while, as expected, living in a household where the individuals were exposed to alcohol abuse or drug use predicted cannabis use at follow-up. The finding that having been in a residential program was associated with abstinence in the last month of treatment, completing treatment, but not being abstinent at follow-up may be related to the benefits of a more structured setting in treatment and the lack of a stable and sober living environment after treatment.

Employment was also an important factor in this study. At admission, coerced clients were more likely to be employed and to report fewer days of employment problems as measured on the ASI employment domain. At follow-up, both coerced and voluntary clients who reported more days of employment problems were more likely to be using cannabis. Likewise, more attendance at 12-step meetings was a significant predictor of treatment completion and abstinence from cannabis in the month preceding treatment discharge and in the month preceding 90-day follow-up.

## Conclusion

This report profiles two types of clients who enter treatment in Texas with a primary problem with cannabis: (1) those who are less impaired who were sent to treatment as a result of their involvement in the criminal justice system and (2) those who were more impaired who entered treatment voluntarily. These findings support the positive treatment outcomes for clients legally coerced into cannabis treatment compared to non-coerced clients. The study also highlights the need for a greater range of interventions to assist those with cannabis use disorders before they develop the range and severity of health and psycho-social problems seen among these non-coerced clients. Early [20,21] and brief [22,23] interventions have been shown to be effective in the management of cannabis use disorders. The raising of public awareness of the prevalence of cannabis use disorders and the availability and efficacy of subsidised, earlier stage and briefer interventions would reduce the burden of these disorders. Screening, detecting and intervening in the early stages of cannabis abuse would reduce the need for more intensive residential services for severely impaired cannabis-dependent individuals.

Given the high rates of physical and psychological health problems and frequency of contact with the emergency rooms, there is an urgent need for routine screening for cannabis use disorders in primary health care settings. Training clinicians in the delivery of these 1–2 session interventions comprising motivational enhancement and cognitive behavioural therapy skills, in a variety of settings including adolescent health services, juvenile justice facilities and primary health care, would further improve cannabis treatment outcomes at earlier stages of the disorder. Those clients with severe psycho-social and health problems associated with their cannabis use should have greater access to subsidised specialist residential treatment services that have expertise in the management of comorbid psychiatric and cannabis use disorders.

While this is a large dataset, it is representative only of lower income clients who entered publicly-funded treatment in Texas. The 90-day follow-up data is largely self-reported and was not biochemically validated and the reporting on DSM-IV diagnoses was not uniform across all programs. The size of the dataset and the large number of variables in BHIPS, however, provides insight into treatment characteristics and the short-term treatment outcomes of those individuals who develop problems due to their cannabis use. This study did not analyze treatment outcome by type of legal coercion employed. Coercion is a complex psychological construct and this study was not able to assess issues such as resistance to treatment, which has been shown to be an important factor in treatment outcome, particularly among those coerced into treatment [24]. Future research should address these limitations.

## Competing interests

The author(s) declare that they have no competing interests.

## Authors' contributions

JC guided aspects of the analyses and was the lead author on the introduction and conclusions sections of the paper. JCM secured access to the data, performed the analyses and was the lead author of the methods, results, and discussion sections. Both authors read and approved the final manuscript.

## Pre-publication history

The pre-publication history for this paper can be accessed here:


